# Enabling and improving *trans*-nerolidol production by *Corynebacterium glutamicum*: combining metabolic engineering and trace elements medium refinement

**DOI:** 10.3389/fbioe.2025.1621955

**Published:** 2025-06-23

**Authors:** Jan Seeger, Stella Lohoff, Fabian Schmitfranz, Nadja A. Henke, Volker F. Wendisch

**Affiliations:** Genetics of Prokaryotes, Faculty of Biology and Center for Biotechnology (CeBiTec), Bielefeld University, Bielefeld, Germany

**Keywords:** *Corynebacterium glutamicum*, *trans*-nerolidol, terpenes, design of experiment, media optimization, metabolic engineering

## Abstract

Terpenes are biomolecules of significant industrial relevance, with applications in pharmaceuticals, cosmetics, and the food industry. Their biotechnological production is emerging, with *Corynebacterium glutamicum*, a Gram-positive bacterium traditionally employed for large-scale amino acid production, serving as a promising host. While metabolic engineering strategies have been extensively applied to enhance terpene titers in *C. glutamicum*, the role of medium composition, particularly trace elements, remains underexplored. In this study, the impact of trace element composition on *trans*-nerolidol production by engineered *C. glutamicum* was investigated. A Design of Experiments (DoE) approach identified MgSO_4_ as a critical factor, and the refined trace element composition led to a 34% increase in *trans*-nerolidol production. Further metabolic engineering efforts resulted in a final titer of 28.1 mg L^-1^. Subsequent fed-batch fermentation achieved a *trans*-nerolidol titer of 0.41 g L^-1^, representing the highest reported sesquiterpene titer being produced by *C. glutamicum* to date. Additionally, the refined trace element composition was successfully applied to patchoulol- and (+)-valencene-producing strains, leading to production increases of 15% and 72%, respectively. These findings demonstrate that trace element refinement and metabolic engineering act as complementary strategies for enhancing terpene production in a microbial production host.

## 1 Introduction

With more than 80,000 characterized molecules, terpenes are the largest and structurally most diverse group of natural products (Christianson, 2017). Fulfilling a myriad of functions in nature, terpenes are, for example, involved in photosynthesis (Zulfiqar et al., 2021), regulation of biotic and abiotic stress (Wang et al., 2023), defense and attraction (Gershenzon and Dudareva, 2007), as well as membrane fluidity (Zhou et al., 2015). Terpenes are also of significant industrial interest as they find application in pharmaceuticals, cosmetics, food, and biofuels (Caputi and Aprea, 2011; Wang et al., 2015; Fan et al., 2023). Most of these compounds have to be extracted from plants, which poses significant challenges due to low concentrations in plant tissues, seasonal and geographical fluctuations, and the extensive agricultural areas required. To avoid this, biotechnological production offers a sustainable and efficient alternative to produce terpenes (Moser and Pichler, 2019; Zhang and Hong, 2020). Among the different microbes available, the Gram-positive industrial workhorse *Corynebacterium glutamicum*, extensively used for the large-scale production of amino acids (Wendisch, 2020), is a promising cell factory for natural compounds as its deriving products are classified as GRAS (Cankar et al., 2023). In recent years, *C. glutamicum* has been engineered for the production of various terpenes including hemi-, mono-, sesqui-, di- and triterpenes (Kang et al., 2014; Henke et al., 2018a; Henke et al., 2018b; Sasaki et al., 2019; Lim et al., 2020; Luckie et al., 2024; Li et al., 2025; Lee et al., 2025). To increase the product titer, mostly metabolic engineering strategies were pursued. Competing pathways like carotenogenesis (Henke et al., 2018b) or in the central carbon metabolism (Li et al., 2025) were deleted. Lim et al. (2020) identified and overexpressed key enzymes of the methylerythritol 4-phosphate (MEP) pathway to enhance precursor supply. In contrast to that, Luckie et al. (2024) and Sasaki et al. (2019) introduced the heterologous mevalonate (MVA) pathway to circumvent endogenous regulation of the MEP pathway that might limit carbon flux. In addition, shake flasks cultivation conditions have been optimized (Li et al., 2025).

Metabolic engineering efforts have to be combined with process intensification: cultivation conditions and the cultivation medium may impact the overall performance of the strain, as the medium can influence cell growth and productivity (Galbraith et al., 2018). Since there is no universal approach for media optimization, different methods have been used. The one-factor-at-a-time (OFAT) experiments might be the most commonly applied technique for media optimization (Singh et al., 2017). Here, only one factor is varied while the other variables are kept constant. This approach was used to investigate the effect of several media additives for a poly (3-hydroxybutyrate) producer (Nikel et al., 2008). Although the OFAT technique is simple and convenient, the large number of experiments are laborious, time consuming, and costly. Furthermore, interactions between the variables cannot be detected and the optimum might be missed completely (Singh et al., 2017). By using statistical approaches like the design of experiments (DoE), the limitations of the OFAT can be overcome. This systematic approach allows to vary multiple parameters simultaneously, thereby, identifying significant variables, their interactions, and optimal conditions with minimal experimental effort (Fisher, 1926). The two-level fractional factorial Plackett-Burman design (PBD) aims to identify major effects while neglecting interactions. Due to the low number of experiments, this experimental design is often applied in early-stage development to identify significant parameters (Singh et al., 2017). PDB has been used for media optimization for enzyme productions like chitinase and β-amylase (Rama et al., 1999; Vaidya et al., 2003). To uncover interactions among the different variables and to determine optimum conditions, response surface methodology (RSM) becomes necessary. Therefore, experimental designs such as the Box-Behnken or central composite design (CCD) are required. The CCD consists of a (fractional) factorial core, star points that extend the design beyond the factorial levels as well as center points (Gündogdu et al., 2016; Singh et al., 2017). In terms of media optimization, CCD and RSM have been successfully used to improve the production of oxytetracycline and actinorhodin (Elibol, 2004; Singh et al., 2012).

The standard cultivation medium for *C. glutamicum* is the CGXII minimal medium (Keilhauer et al., 1993). Initially established for amino acid production, the components have been adapted to different production scenarios (Jeon et al., 2013; Hoffmann and Altenbuchner, 2014; Buchholz et al., 2014; Ko et al., 2018). To date, there is no medium dedicated for the production of terpenes by *C. glutamicum*. Variations in glucose concentration and C:N ratio have been investigated for isopentenol production, but exhibited effects comparable to standard CGXII conditions (Sasaki et al., 2019). Consequently, other media components might be worth to be investigated. Given that trace elements, despite their low concentrations, have been shown to enhance the production of l-lysine and carotenoids in *C. glutamicum* (Weuster-Botz et al., 1997; Meyer et al., 2025), as well as surfactin in *Bacillus subtilis* (Wei et al., 2007), xylitol in *Debaryomyces hansenii* (Bustos Vázquez et al., 2017), proteins in *Pichia pastoris* (Isidro et al., 2016; Tavasoli et al., 2017), and lipids and citric acid in *Yarrowia lipolytica* (Kumar et al., 2021), their potential to improve terpene biosynthesis should be further explored.

Therefore, the focus of this study was a DoE-based approach to optimize the trace element composition of CGXII medium for the production of terpenes with *C. glutamicum*. To study the effects of the trace elements on terpene production, *trans*-nerolidol was chosen as a model terpene. This sesquiterpene is used in decorative cosmetics like perfumes and shampoos as well as non-cosmetic products such as household cleansers and has been approved by the U.S. Food and Drug Administration as food flavoring agent (Chan et al., 2016). Furthermore, nerolidol exhibits antioxidant (Zhao et al., 2020), antitumor (Ambrož et al., 2015), and antidiabetic (Jiang and Zhang, 2022) activities, making it interesting for the pharmaceutical industry. Metabolic engineering was combined with media optimization to improve the overall production titer. In addition, the transferability of the refined trace elements was tested with patchoulol and (+)-valencene.

## 2 Materials and methods

### 2.1 Bacterial strains and growth conditions

Strains and plasmids used in this study are listed in Table 1. *Escherichia coli* was used as cloning host and was cultivated in lysogeny broth (LB (Bertani, 1951)) at 37°C and 180 rpm. *C. glutamicum* was used for production experiments. Precultures were cultivated in 100 mL baffled shake flasks containing LB supplemented with 10 g L^-1^ glucose at 30°C and 120 rpm. The pre-cultivated cells were used to inoculate the main culture to an initial optical density (wavelength: 600 nm, OD_600_) of 1 determined by using the V-1200 spectrophotometer (VWR, Radnor, PA, United States). All *trans*-nerolidol producing experiments, including response surface methodology (see Section 2.3), were performed in 48-well FlowerPlates (Beckman Coulter, Brea, CA, United States) with a filling volume of 1 mL per well at 1,100 rpm and 30°C in the BioLector XT microcultivation system (Beckman Coulter, Brea, CA, United States) for 24 h. For the transfer of the refined trace elements to patchoulol and (+)-valencene, 100 mL baffled shake flasks with a filling volume of 10 mL and additional 10% (v v^−1^) dodecane as organic overlay were used instead of the BioLector. Cultivation lasted 48 h at 30°C and 120 rpm. 40 g L^-1^ glucose was added to the CGXII medium as carbon and energy source (Keilhauer et al., 1993) for all main cultures. If appropriate, the medium was supplemented with kanamycin (25 μg mL^-1^), tetracycline (5 μg mL^-1^), chloramphenicol (7.5 μg mL^-1^ for *C. glutamicum* or 30 μg mL^-1^ for *E. coli*). 1 mM isopropyl-β-D-1-thiogalactopyranoside (IPTG) was added at the start of the main cultivation to induce gene expression.

**TABLE 1:** Strains and plasmids used in this work.

Strain/plasmid	Relevant characteristics	Reference
Strains		
*E. coli* DH5α	F^−^ *thi*-1 *endA1 hsdR17* (r^−^ m^−^) *supE44* Δ*lacU169* (Φ80*lacZ*ΔM15) *recA1 gyr96 relA1*	Hanahan (1983)
*C. glutamicum* WT	Wild type, ATCC 13032	Kinoshita et al. (1957)
Δ*crtOP*Δ*crtB2I’I2*Δ*idsA*	Δ*crtOP* (cg0717-cg0723), Δ*crtB2I’I2* (cg2688-cg2672) Δ*idsA* (cg23843) deletion mutant of ATCC 13032	Henke et al. (2018)
NERO1	ATCC 13032 (pECXC99E-*ispA*_*Ec*_-*NS*_*Tw*_)	This work
NERO2	Δ*crtOP*Δ*crtB2I’I2*Δ*idsA* (pECXC99E-*ispA*_*Ec*_-*NS*_*Tw*_)	This work
NERO3	Δ*crtOP*Δ*crtB2I’I2*Δ*idsA* (pECXT-P_*syn*_-*ispA*_*Ec*_-*NS*_*Tw*_)	This work
NERO4	Δ*crtOP*Δ*crtB2I’I2*Δ*idsA* (pECXT-P_*syn*_-*ispA*_*Ec*_-*NS*_*Tw*_) (pVWEx1)	This work
NERO5	Δ*crtOP*Δ*crtB2I’I2*Δ*idsA* (pECXT-P_*syn*_-*ispA*_*Ec*_-*NS*_*Tw*_) (pVWEx1-*dxs*-*idi*)	This work
PAT3	Δ*crtOP*Δ*crtB2I’I2*Δ*idsA* (pECXT-*ispA*-*PcPS*) (pVWEx1-*dxs*-*idi*)	Henke et al. (2018)
VLC6	Δ*crtE* Δ*idsA* (pEKEx3-*ispA*-*oCNVS*) (pVWEx1-*dxs*-*idi*)	Binder et al. (2016)
Plasmids		
pECXC99E	Cm^R^, *E. coli*/*C. glutamicum* shuttle vector, P_trc_, *lacI* ^*q*^, pGA1 *oriV*_*Cg*_	Kirchner and Tauch (2003)
pECXT-P_*syn*_	Tet^R^, pECXT99A derivative for constitutive expression from synthetic P_*syn*_ promoter	Henke et al. (2021)
pECXC99E-*ispA*_*Ec*_-*NS*_*Tw*_	pECXC99E derivative for the inducible overexpression of *ispA* from *E. coli* and *NS* from *T. wilfordii*	This work
pECXT-P_*syn*_-*ispA*_*Ec*_-*NS*_*Tw*_	pECXT-P_*syn*_ derivative for the overexpression of *ispA* from *E. coli* and *NS* from *T. wilfordii*	This work
pVWEx1-*dxs*_*Cg*_-*idi*_*Cg*_	pVWEx1 derivative for the inducible overexpression of *dxs* and *idi* from *C. glutamicum*	Binder et al. (2016)

### 2.2 Molecular biological techniques

Primers for DNA amplification and sequencing (Supplementary Table S1) were purchased from Sigma-Aldrich (St. Louis, MO, United States). Plasmid DNA isolation (QIAwave Plasmid Miniprep, Qiagen, Venlo, Netherlands) and PCR clean-up (NucleoSpin^®^ Gel and PCR Clean-up, Macherey-Nagel, Düren, Germany) were performed according to the manufacturer’s instructions. Nerolidol synthase (NS) gene from *Tripterygium wilfordii* (GenBank: KU588405) (Su et al., 2017) was codon optimized (Codon Optimization Tool, 2025) and synthesized by Twist Bioscience (San Francisco, CA, United States). Gene fragments were amplified using the Allin™ HiFi DNA polymerase (highQu GmbH, Kraichtal, Germany) and cloned into BamHI-digested (Thermo Fisher Scientific, Waltham, MA, United States) plasmids using Gibson Assembly (Gibson et al., 2009). CaCl_2_ chemocompetent *E. coli* DH5α were transformed with the Gibson Assembly reaction mix by heat shock at 42°C (Sambrook and Russell, 2001). Cloned DNA fragments were verified by DNA sequencing. *Trans*-Nerolidol producing *C. glutamicum* strains were created by transforming electrocompetent cells via electroporation and subsequent heat shock at 46°C with the respective plasmids (Eggeling and Bott, 2005).

### 2.3 Design of experiment and response surface methodology

Cultivation was performed as described in Section 2.1. Due to the total amount of 1 mL per well, the trace elements were prepared separately as 100x stock solutions to facilitate pipetting. Design and analysis of the experiment was performed by using R 4.4.1 (R Studio Team 2024) and the rsm package version 2.10.5 (Lenth, 2009). Within the experiment, the effect of the trace elements MgSO_4_, CaCl_2_, FeSO_4_, MnSO_4_, and ZnSO_4_ on the *trans*-nerolidol titer was investigated. The central composite design consisted of a 2^5^ full factorial design as cube portion, ten face-centered star points and six center points. The concentration of each trace element at the center point was regarded as 100%. 50% and 150% of the center point concentration were chosen for the cube portion of the model. For the star points, only one out of the five trace elements was set to either 0% and 200%, respectively, while the others remained at 100%. The overall workflow was: first CCD with standard CGXII trace elements as center points, followed by a steepest ascent experiment. The best trace elements composition of the steepest ascent experiment was used as the center point for the second CCD. All combinations and volumes can be found in the Supplementary Material. The other media components remained constant as described in Section 2.1.

### 2.4 Terpene extraction and quantification

For the extraction of *trans*-nerolidol, 1 mL of hexane was added to 330 µL of cultivation broth and incubated at 50°C and 1,000 rpm for 30 min (ThermoMixer C, Eppendorf, Hamburg, Germany). The extraction mixture was centrifuged, the organic phase was removed, dried with sodium sulphate and transferred to GC-MS analysis. For patchoulol and (+)-valencene, the dodecane overlay was separated from the aqueous phase by centrifugation and was directly used for analysis. All terpenes were quantified using a TRACE GC Ultra gas chromatograph and a ISQ single quadrupole mass spectrometer equipped with an AS 3000 autosampler and a TraceGOLD™ TG-5 MS column (30 m × 0.25 mm x 0.25 µm) (Thermo Fisher Scientific, Waltham, MA, United States). 1 μL was injected in splitless mode. Helium was used as carrier gas at a constant flow of 1 mL min^-1^. Temperatures were set as the following: injector (250°C), interface (250°C), and ion source (220°C). The oven profile was set as the following: 80°C for 1 min, increased to 120°C at a rate of 10°C min^-1^, followed by 3°C min^-1^–160°C, and a further increase at 10°C min^-1^–270°C, which was held for 2 min. Mass spectra were recorded after a solvent cutoff at 14 min using a scanning range of 50–750 m*/z* at 20 scans s^-1^. Chromatograms were evaluated using Xcalibur 2.1.0 (Thermo Fisher Scientific, Waltham, MA, United States). Extracted-ion chromatograms at *m*/*z* = 93 were used for quantification of *trans*-nerolidol, *m*/*z* = 138 and *m*/*z* = 220 for patchoulol, and *m*/*z* = 161 for (+)-valencene. Analytical standards for *trans*-nerolidol, patchoulol, and (+)-valencene were purchased from Extrasynthese (Lyon, France), Biosynth (Staad, Switzerland), and Sigma-Aldrich (St. Louis, MO, United States), respectively.

### 2.5 Bioreactor fed-batch fermentation

Fed-batch fermentation using strain NERO5 was performed in a bioreactor with a total volume of 3.7 L (KLF, Bioengineering AG, Wald, Switzerland). The aspect ratio of the reactor was 2.6:1.0. Three six-bladed Rushton turbines were placed along the stirrer axis at 6, 12, and 18 cm from the bottom of the reactor with a stirrer to reactor diameter ratio of 0.39. 1 L of the high cell density medium based on Knoll et al. (2007) was used with some alterations: all trace elements were replaced by the refined trace elements in a 20x excess compared to the regular cultivation in CGXII (see Supplementary Table S2). Antibiotics and IPTG were added accordingly and the medium was inoculated to an OD_600_ of 1. 500 mL of a 600 g L^-1^ glucose solution was used as feed medium. A headspace overpressure of 0.5 bar was applied. The temperature was kept at 30°C during fermentation. The pH of 7 was automatically maintained by the addition of 10% (v v^−1^) H_3_PO_4_ and 25% (v v^−1^) NH_3_. An initial airflow of 0.25 NL min^-1^ was provided from the bottom through a ring sparger, which was manually increased during the fermentation to 0.75 NL min^-1^ when oxygen supply became limiting. The stirrer speed increased automatically in a stepwise manner from 400 rpm to 1,500 rpm, every time the relative dissolved oxygen saturation (rDOS) fell below 30%. The feed pump was primed when the rDOS fell below 60% for the first time. Subsequently, the feed pump was activated every time the rDOS exceeded 50% and stopped as soon as the rDOS fell below 50% thereby preventing overfeeding. To control foam formation, 0.6 mL L^-1^ of antifoam 204 was already added to the batch medium. If required, an antifoam probe controlled the supply of antifoam 204 during the process. Samples were automatically taken over the course of the fermentation and stored at 4°C until further use.

## 3 Results

### 3.1 Establishment of *trans*-nerolidol production in *C. glutamicum*

**FIGURE 1:**
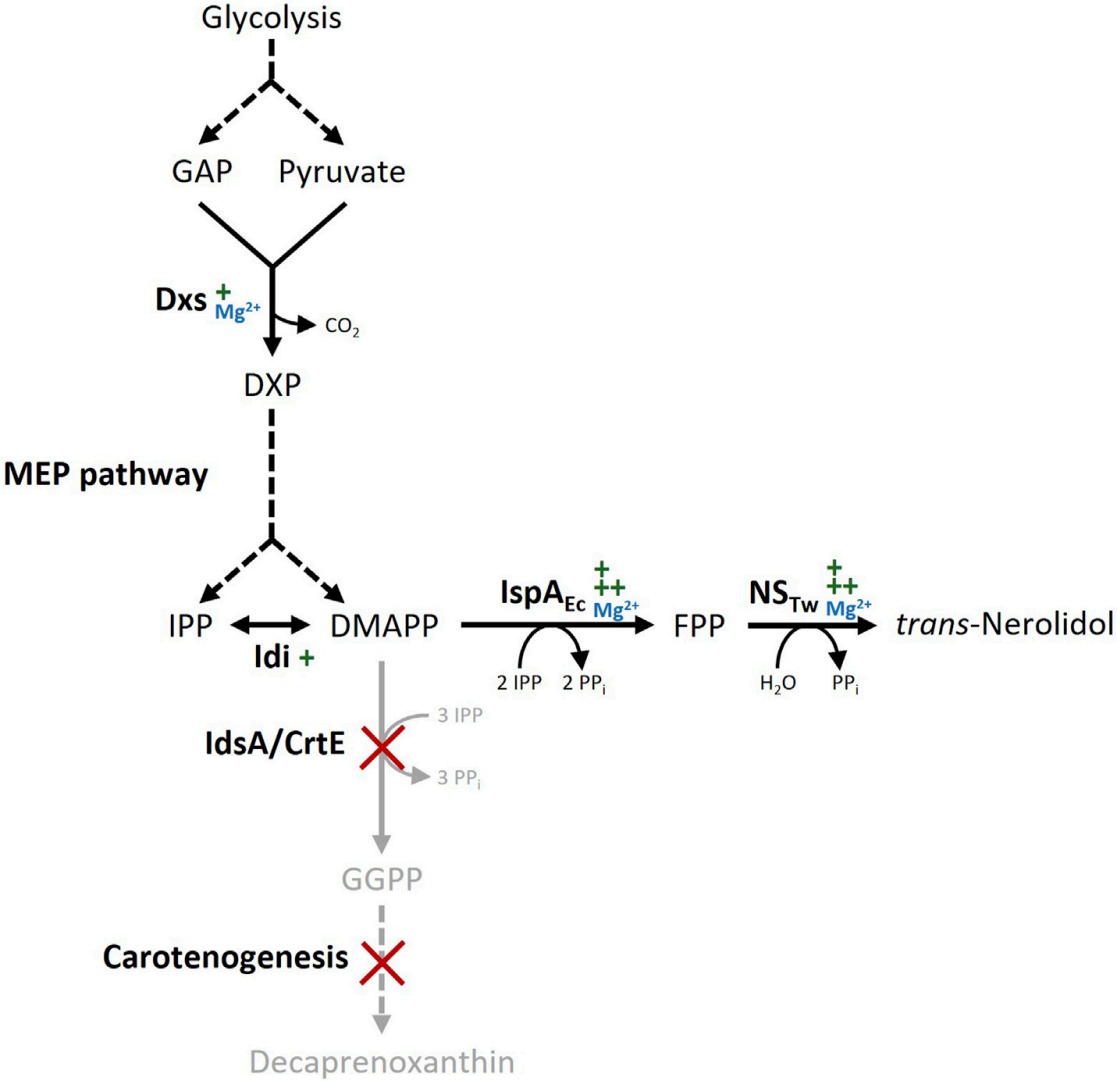
Biosynthetic pathway for *trans*-nerolidol production in *Corynebacterium glutamicum*. Dashed arrows represent multiple enzymatic steps, gene deletions are marked with red crosses, Mg^2+^ dependency of key enzymes is indicated in blue. Gene overexpression is indicated by green plus symbols; the number of symbols refers to expression strength. Abbreviations: GAP, glyceraldehyde 3-phosphate; DXP, 1-deoxy-D-xylulose 5-phosphate; IPP, isopentenyl pyrophosphate; DMAPP, dimethylallyl pyrophosphate; FPP, farnesyl pyrophosphate; GGPP, geranylgeranyl pyrophosphate; DXS, DXP synthase; MEP, 2-*C*-methyl-D-erythritol 4-phosphate; Idi, IPP isomerase; IspA_Ec_, FPP synthase from *Escherichia coli*; NS_Tw_, Nerolidol synthase from *Tripterygium wilfordii*; IdsA, GGPP synthase; CrtE, GGPP synthase.

**FIGURE 2:**
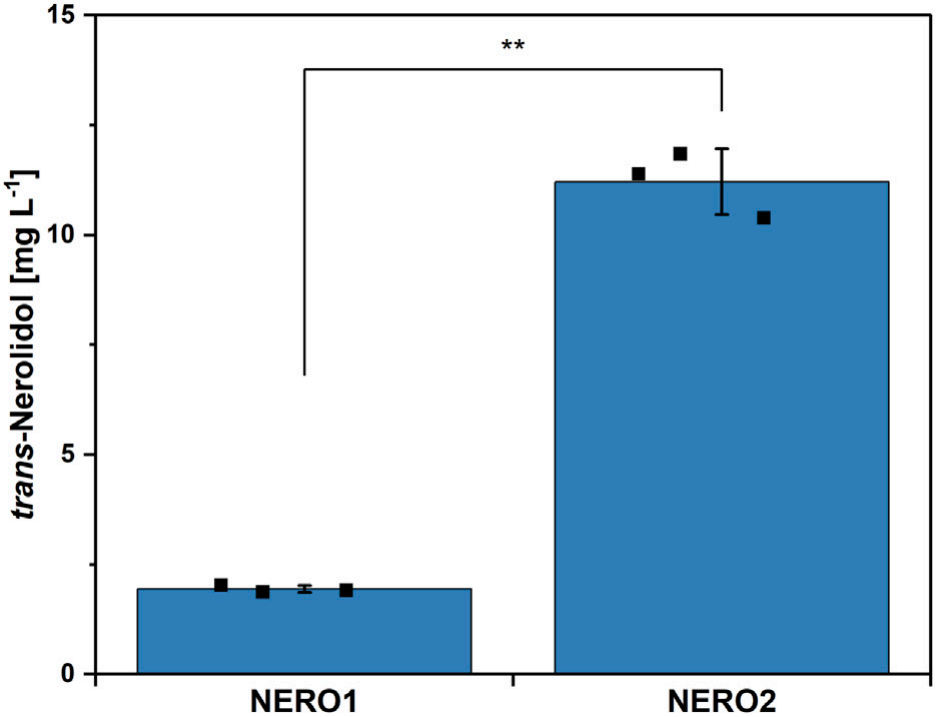
*trans*-Nerolidol production by *C. glutamicum* NERO1 and NERO2. Means, standard deviations as error bars, and single points of triplicate cultivations are given. Statistical significance was calculated with a Students’ t-test p < 0.05 (*), p < 0.01 (**), p < 0.005 (***).

The genome of *C. glutamicum* codes for two geranylgeranyl pyrophosphate (GGPP) synthases (*crtE* and *idsA*), but neither of them synthesizes significant amounts of the sesquiterpene precursor farnesyl pyrophosphate (FPP) from the end products of the MEP pathway isopentenyl pyrophosphate (IPP) and dimethylallyl pyrophosphate (DMAPP) (Frohwitter et al., 2014). *Trans*-Nerolidol production in *C. glutamicum* wild type was enabled by overexpressing FPP synthase *ispA* from *E. coli* together with a codon-optimized nerolidol synthase from *T. wilfordii* from the plasmid pECXC99E (NERO1, see Figures 1, 2). To prevent further conversion of FPP to GGPP and subsequently to carotenoids, a metabolically engineered wild type derivative lacking both carotenoid operons as well as the GGPP synthase *idsA* (Henke et al., 2018b) was transformed with the plasmid pECXC99E-*ispA*_*Ec*_-*NS*_*Tw*_ (NERO2). This strain did not synthesize carotenoids, thus increasing the *trans*-nerolidol titer 5.9-fold from 1.9 ± 0.1 to 11.2 ± 0.8 mg L^-1^. The engineered *C. glutamicum* strain NERO2 was subsequently used to study the effects of the trace element composition.

### 3.2 Design of experiments for refinement of trace element composition

Although some media formulations for *C. glutamicum* contain additional trace elements like Na_2_MoO_4_ and H_3_BO_3_ (Weuster-Botz et al., 1997), the classical CGXII medium contains the seven trace elements MgSO_4_, CaCl_2_, FeSO_4_, MnSO_4_, ZnSO_4_, CuSO_4_, and NiCl_2_ (Keilhauer et al., 1993). To reduce the number of experiments, it was decided to neglect the two trace elements with the lowest concentration, being CuSO_4_ and NiCl_2_. This is in accordance with the fact that out of the seven trace elements in CGXII, copper and nickel ions are the metal ions used least as cofactors in enzymes (Waldron et al., 2009). Therefore, it was hypothesized that variation of the copper and nickel ion concentrations might influence terpene biosynthesis the least. A CCD approach, with the standard CGXII trace elements concentrations (see Supplementary Table S3) as center points, was performed in the BioLector microcultivation system using strain NERO2 (see Section 2.3).

The analysis of the *trans*-nerolidol titer showed no significant two-factor interactions between the trace elements (data not shown). This is why the data was subsequently evaluated considering only first order and quadratic effects. Significant first-order effects of MgSO_4_ as well as quadratic effects of MgSO_4_ and FeSO_4_ were observed (see Supplementary Table S4). Although the model possessed no significant lack of fit, the quadratic effects might be just artificial as the star points containing no MgSO_4_ or FeSO_4_ did not grow and therefore did not produce any *trans*-nerolidol (see boxplots in Supplementary Figure S1 and Supplementary Table S3). This is why only first order effects were considered, showing strong positive effects of MgSO_4_ (Table 2).

**TABLE 2:** Effects of the trace elements on *trans*-nerolidol titer considering first order effects. The effect of each factor, the F-values and their probabilities are given.

Factor	*t*-value	Prob > *t*
Intercept	25.25	<0.001
FeSO_4_	0.6923	0.4926
MnSO_4_	0.8497	0.4003
ZnSO_4_	−0.314	0.7551
CaCl_2_	−0.8093	0.4229
MgSO_4_	7.27	<0.001

	F-value	Prob > F
First Order	10.97	<0.001
Lack of fit	4.57	0.05

**FIGURE 3:**
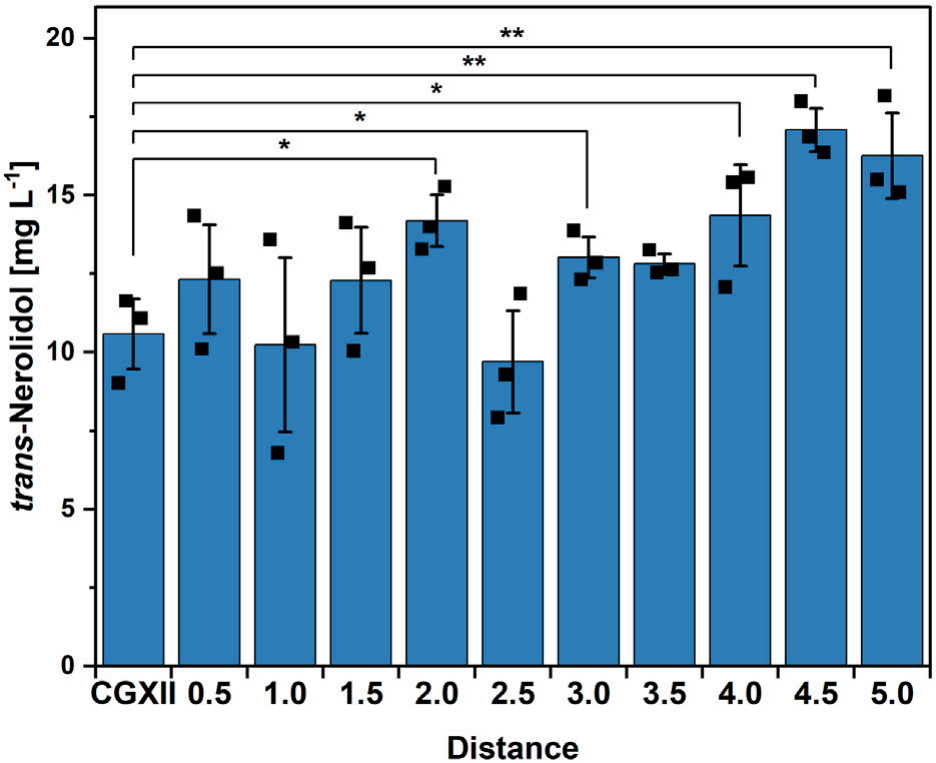
Steepest ascent experiment based on the first CCD. Means, standard deviations as error bars, and single points of triplicate cultivations are given. Statistical significance was calculated with a Students’ t-test p < 0.05 (*), p < 0.01 (**), p < 0.005 (***).

By applying only a first order model, no stationary point with a predicted optimum could be obtained. Furthermore, it was expected to gain more insight into the interactions between the different trace elements and their effects on *trans*-nerolidol production. A reasonable next step therefore is to follow the direction along where the response increases the fastest to end up at trace element concentrations that result in higher titers than the starting condition. This position would be the new center point used for a second round of CCD (Roberts et al., 2020). The so-called path of steepest ascent was calculated by the results of the first order model out of the CCD, yielding a set of trace element compositions and predicted titers (see Supplementary Table S5). The results of the steepest ascent analysis are shown in Figure 3. Although fluctuating, a trend of increasing production along the path of steepest ascent was observed. At a distance of 4.5, the highest titer (17.1 ± 0.7 mg L^-1^) was observed, being significantly higher than the control. This trace element composition was subsequently used as a new center point for another CCD, allowing to investigate concentration ranges that might be closer to an optimum (see Supplementary Table S6). After having performed the second CCD experiment, neither two-factor interactions, quadratic effects or first order models could be fitted significantly (data not shown).

### 3.3 Verification of trace element composition

**FIGURE 4:**
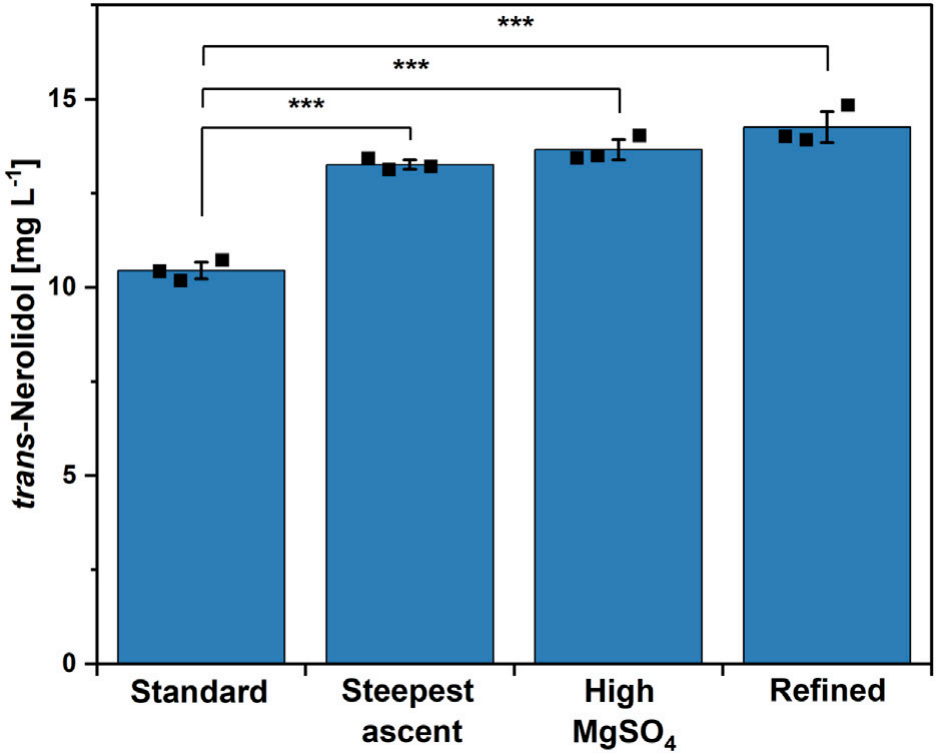
Comparison of different trace element compositions using strain NERO2. From left to right: standard CGXII trace elements, best composition of steepest ascent experiment, standard CGXII trace elements with 4x MgSO_4_ concentration, best composition of second CCD experiment (=refined). Means, standard deviations as error bars, and single points of triplicate cultivations are given. Statistical significance was calculated with a Students’ t-test p < 0.05 (*), p < 0.01 (**), p < 0.005 (***).

The second round of CCD did not result in a significant result. However, some of the tested trace element combinations within this new range of concentration showed a clearly increased titer compared to the control. To validate these observations, the best composition from the second CCD (see Supplementary Table S6, run 45) was compared to the standard trace elements and to the best combination of the steepest ascent experiment, which was used as the center point of the second CCD. As MgSO_4_ showed a strong positive effect in the first CCD (Table 2), the influence of a 4x increased MgSO_4_ concentration was investigated as well. The tested trace element compositions are listed in Supplementary Table S2 and the results are shown in Figure 4. All three trace element compositions resulted in significantly increased titers. The highest titer of 14.3 ± 0.4 mg L^-1^, corresponding to an improvement of 34%, was achieved using the trace element composition found within the second CCD. No statistical significance was found when comparing the high MgSO_4_ composition with the one from the second CCD. However, as the overall amount of trace elements changed the least when compared to the other compositions (see Supplementary Table S2), the combination obtained within the second CCD was now chosen to be the refined trace element combination.

### 3.4 Improved gene overexpression of terminal biosynthesis enzymes and of MEP pathway genes

Trace element refinement improved *trans*-nerolidol production of the base strain NERO2. To answer the question, if the refined trace elements would also support higher *trans*-nerolidol production, strain NERO2 was further metabolically engineered (Figure 1) before standard and refined media were compared.

**FIGURE 5:**
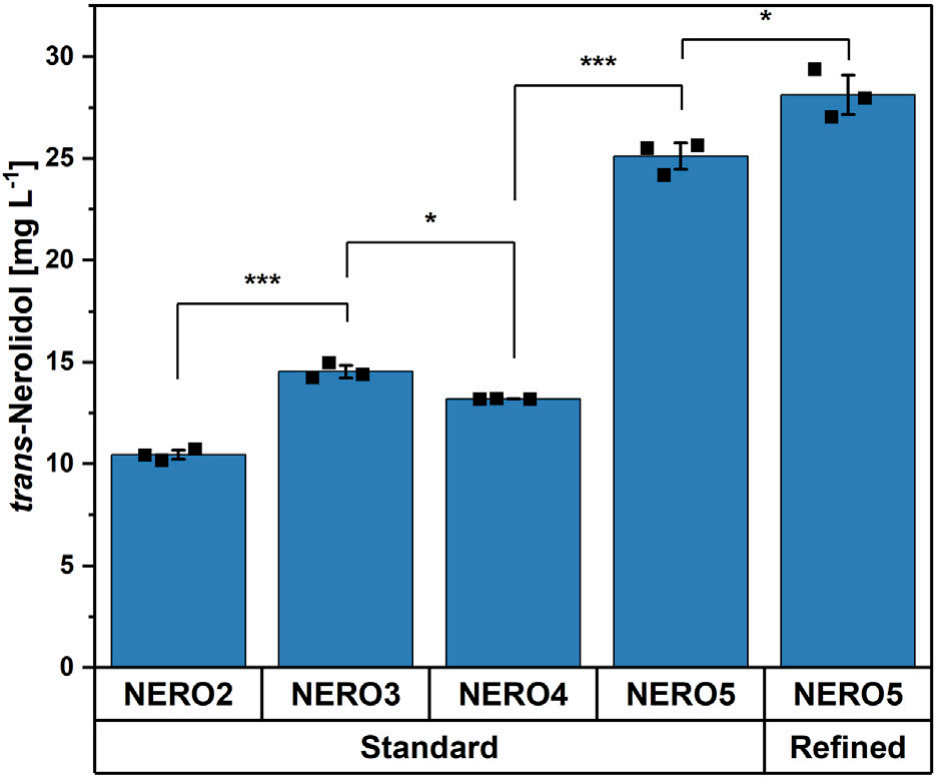
Comparison of different *trans*-nerolidol producing strains with standard and refined trace elements. Means, standard deviations as error bars, and single points of triplicate cultivations are given. Statistical significance was calculated with a Students’ t-test p < 0.05 (*), p < 0.01 (**), p < 0.005 (***).

To improve the terminal biosynthesis, the synthetic operon of FPP synthase and *trans*-nerolidol synthase genes was cloned into the pECXT-P_syn_ vector containing the strong constitutive P_syn_ promoter (Henke et al., 2021) yielding strain NERO3. Using the standard trace elements, the stronger expression increased the titer significantly to 14.5 ± 0.3 mg L^-1^ (compare strains NERO2 and NERO3 in Figure 5). The addition of the empty vector pVWEx1 to NERO3 (NERO4; empty vector control) slightly decreased the titer. To improve the entry reaction into the MEP pathway as well as the interconversion of IPP and DMAPP, *dxs* and *idi* were overexpressed as a synthetic operon (NERO5) resulting in a titer of 25.1 ± 0.7 mg L^-1^, which was 2.4 times higher compared to strain NERO2 (Figure 5). Next, it was tested, if the refined traces would have a positive impact on *trans*-nerolidol production by strain NERO5. Indeed, the refined trace elements further significantly improved *trans*-nerolidol production to the titer to 28.1 ± 1.0 mg L^-1^ (+12%).

### 3.5 Bioreactor fed-batch fermentation

**FIGURE 6:**
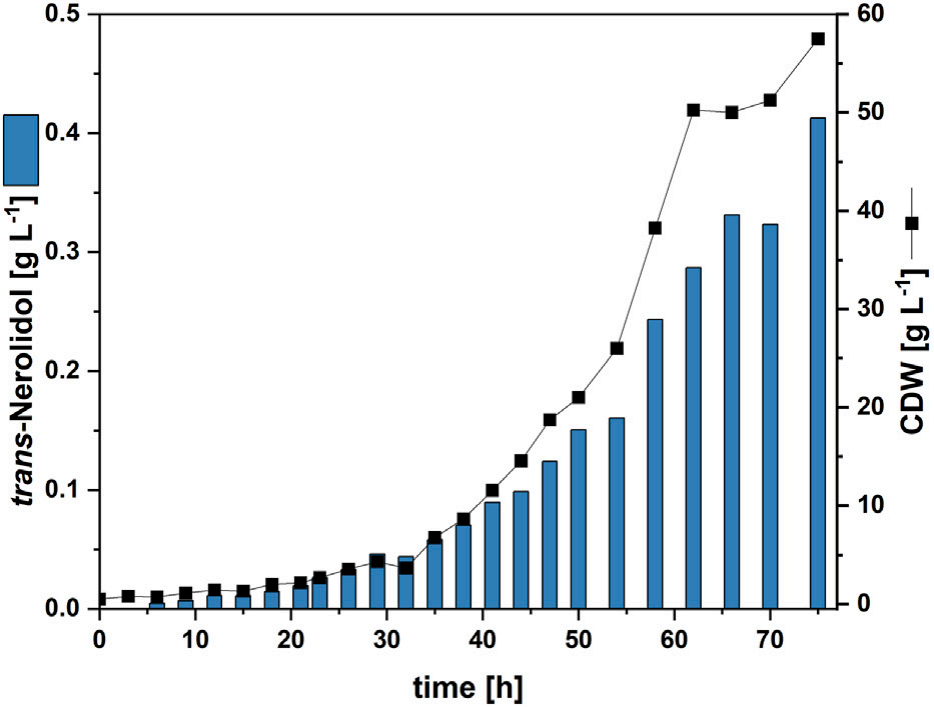
Fed-batch fermentation of NERO5. Cell dry weight concentration (CDW) and *trans*-nerolidol titer are given.

Since the combination of trace element refinement and metabolic engineering was successful in a microcultivation system, it was evaluated whether the *trans*-nerolidol production using *C. glutamicum* strain NERO5 can be stably transferred to a 1.5 L fed-batch process, corresponding to a 1,500-fold volume increase. The high cell density medium based on Knoll et al. (2007) was used for fermentation. However, the trace element composition of this medium corresponds neither to the standard CGXII nor to the refined trace elements. Hence, the refined trace element composition was used instead, while all other media components remained unchanged. To account for the higher cell density, the concentration of refined trace elements was increased proportionally 20-fold relative to standard CGXII cultivations in BioLector or shake flasks (see Supplementary Table S2). The glucose feed was consumed after 74 h resulting in a biomass titer of 57.5 g_CDW_ L^-1^. The *trans*-nerolidol titer reached 0.41 g L^-1^, corresponding to a volumetric productivity of 5.6 mg L^-1^ h^-1^ (Figure 6).

### 3.6 Transfer of refined trace elements to the production of other sesquiterpenes

**FIGURE 7:**
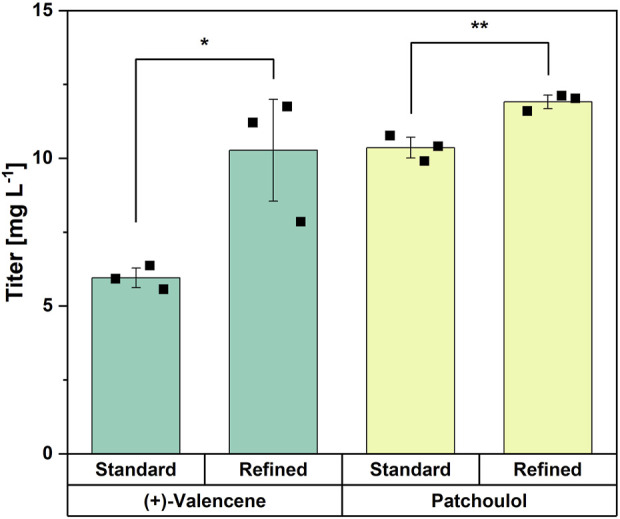
Comparison of standard CGXII with refined trace elements for the production of (+)-valencene and patchoulol. Means, standard deviations as error bars, and single points of triplicate cultivations are given. Statistical significance was calculated with a Students’ t-test p < 0.05 (*), p < 0.01 (**), p < 0.005 (***).

To test if the refined medium can be transferred as a general strategy for the production of other sesquiterpenes, it was used to cultivate previously established strains overproducing patchoulol (Henke et al., 2018b) and (+)-valencene (Binder et al., 2016). The refined trace elements increased the (+)-valencene titer from 6.0 ± 0.3 mg L^-1^ using standard CGXII trace elements to 10.3 ± 1.7 mg L^-1^, corresponding to a 72% improvement. Also, the patchoulol titer was increased, i.e., from 10.4 ± 0.4 mg L^-1^ to 11.9 ± 0.2 mg L^-1^, corresponding to a 15% improvement (Figure 7). Thus, the medium with refined trace elements developed for *trans*-nerolidol production also significantly improved production of two other sesquiterpenes.

## 4 Discussion

In this study, *trans*-nerolidol production in *C. glutamicum* was enabled, thereby expanding the repertoire of C15 terpenes beyond the previously reported compounds patchoulol (Henke et al., 2018b) (+)-valencene (Binder et al., 2016), and α-farnesene (Lim et al., 2020). To improve its production a combined approach of metabolic engineering and media refinement was chosen which increased the product titer about 15 fold from 1.9 ± 0.1 to 28.1 ± 1.0 mg L^-1^. Significant improvements by applying the refined trace elements were also demonstrated for the production of patchoulol and (+)-valencene.

Instead of employing an OFAT approach to investigate the effects of the trace element composition on *trans*-nerolidol production, a DoE methodology was implemented, reducing the number of experiments required while simultaneously enabling the investigation of interactions. In an initial round of experiments, MgSO_4_ was identified as a significant positive factor influencing *trans*-nerolidol production. A subsequent steepest ascent experiment was followed by a second round of CCD, which did not yield statistically significant results. This outcome might be associated with broad concentration ranges in the experimental design. While magnesium, iron, and zinc are essential for the growth of *C. glutamicum*, the remaining trace elements exhibit less pronounced effects (Nakayama et al., 1964; Yang et al., 2021; Meyer et al., 2025). To improve future studies, experimental conditions should be chosen to avoid complete growth inhibition. Nevertheless, the positive effect of MgSO_4_ was confirmed by using a high MgSO_4_ medium.

In general, terpene synthases require divalent cations, typically Mg^2+^, as cofactors (Rudolf and Chang, 2020). Some terpene synthases are also known to use alternative metal cofactors such as manganese or iron (Steele et al., 1998). The metal cofactor mediates the abstraction of the diphosphate, thereby creating a reactive carbocation, and additionally neutralizes the diphosphate over the course of the reaction (Vattekkatte et al., 2018). In the absence of an divalent metal ion, terpene synthases are not active (Degenhardt and Gershenzon, 2000). However, it is crucial for the activity of the terpene synthase, that the correct metal cofactor is bound, which has been shown for magnesium and manganese particularly (Steele et al., 1998; Whitehead et al., 2023). Strong Mg^2+^ dependency was shown for the *trans*-nerolidol synthase used in this study (Su et al., 2017). Since unbound metals compete for a binding site, a phenomenon known as mismetalation can occur, leading to reduced enzyme activity (Foster et al., 2022). It was shown for the *trans*-nerolidol synthases from maize and kiwifruit that Mg^2+^ supported the highest activity, whereas Mn^2+^ or Zn^2+^ diminished activity (Degenhardt and Gershenzon, 2000; Schnee et al., 2002; Green et al., 2012). Hence, it can be hypothesized that a refined trace element composition, with increased Mg^2+^, but reduced Mn^2+^, Fe^2+^, and Zn^2+^, mitigated unfavorable mismetalation, thereby enhancing enzymatic activity and increasing terpene titers. This favorable ratio was successfully achieved either by supplying a high surplus of Mg^2+^ (high MgSO_4_ medium) or by moderately increasing Mg^2+^ while reducing Mn^2+^, Fe^2+^, and Zn^2+^ concentrations (refined medium). An OFAT approach might have resulted in a similar result as obtained for the high MgSO_4_ medium. However, the specific combination of an elevated magnesium ion concentration along the reduction of other components was only identifiable through a DoE-based approach, highlighting the advantage of this methodology over conventional optimization strategies.

Although the terpene synthases expressed in the patchoulol- and (+)-valencene-producing strains have been shown to utilize Mg^2+^ as a cofactor (Munck and Croteau, 1990; Sharon-Asa et al., 2003), the refined trace element composition resulted in varying degrees of improvement, potentially related to differences in cofactor affinity and preferences among the enzymes. Besides terpene synthases, other enzymes involved in terpene biosynthesis may also benefit from increased Mg^2+^ availability, supporting the broader applicability of the refined trace element formulation for the production of diverse terpene compounds. Dxs, which catalyzes the rate-limiting step of the MEP pathway and, thus, supply of the precursor substrates IPP and DMAPP, requires Mg^2+^ (Xiang et al., 2007). Enhanced Mg^2+^ availability may therefore contribute to an overall increase in MEP pathway flux. In addition, FPP synthase *ispA* from *E. coli*, which was heterologously overexpressed in this study as well as in the patchoulol- and (+)-valencene-producing strains, was also shown to utilize Mg^2+^ as a cofactor (Hosfield et al., 2004). Recently, the importance of zinc ions for α-bisabolene production in *Rhodosporidium toruloides* was shown, which was associated with its role as a cofactor for the isopentenyl diphosphate isomerase and in lipid synthesis (Adamczyk et al., 2025). However, magnesium was not investigated in this study (Adamczyk et al., 2025).

In terms of metabolic engineering, three steps were applied in this study to enhance *trans*-nerolidol biosynthesis. First, a strain lacking the carotenogenesis was selected to prevent conversion of FPP to GGPP and onwards to carotenoids. In the second step, two expression levels of the terminal biosynthetic enzymes were evaluated, since terpene synthase activity is often the rate-limiting step under high-flux conditions (Whitehead et al., 2023). In addition to the enhanced enzymatic activity facilitated by the optimized trace element composition, the stronger expression showed a positive effect on *trans*-nerolidol titer. Instead of solely increasing the expression strength, the application of a translational fusion of FFP synthase and NS was successfully shown to enhance nerolidol production in yeast (Cheah et al., 2023). Additionally, the choice of the synthase is important as *trans*-nerolidol synthases exhibit considerable variability in activity (Tan et al., 2023), which can be further improved by enzyme engineering (Liu et al., 2022). Furthermore, the overexpression of *dxs* and *idi*, encoding the key enzymes of the MEP pathway, almost doubled the product titer. Previous studies have demonstrated further MEP pathway optimization, such as the overexpression of *ispD* and *ispF* or *dxr* along with *dxs* and *idi* (Lim et al., 2020). However, no universal approach has yet been identified, as the most effective target genes vary depending on the specific terpene product making additional fine-tuning necessary (Lim et al., 2020). Although not as prominent as the 34% increase for the basic strain NERO2, the refined trace elements still achieved a significant 12% improvement when applied for the engineered strain NERO5, underlining the importance of combined strain and media optimization.

Strain NERO5, in combination with the refined trace element composition, was subsequently implemented in a fed-batch fermentation process, yielding 0.41 g L^-1^ *trans*-nerolidol. As so far 60 mg L^-1^ of patchoulol were produced in a fed-batch fermentation by *C. glutamicum* (Henke et al., 2018b), the process shown here reached the highest titer of a sesquiterpene produced by *C. glutamicum* reported to date. In contrast, 16 g L^-1^, 4.2 g L^-1^, and 11.1 g L^-1^ of *trans*-nerolidol have been produced by *E. coli, Saccharomyces cerevisiae, and Y. lipolytica,* respectively. (Liu et al., 2022; Cheah et al., 2023; Tan et al., 2023). Given that the refined trace element composition appears to influence multiple steps within the terpene biosynthetic pathway and its applicability to the production of other sesquiterpenes, existing terpene-producing strains may benefit from medium adaptation.

Instead of using a chemically defined glucose-based medium as used in this study, alternative substrates could be employed. *C. glutamicum* was shown to grow on a variety of complex substrates derived from agro-industrial side streams, including residues from wheat processing and aquaculture as well as hydrolysates from rice straw, oat spelts, orange peel and hazelnut husk (Buschke et al., 2011; Burgardt et al., 2021; Sasikumar et al., 2021; Pakalın et al., 2023; Schmitt et al., 2023; Junker et al., 2024). Notably, some of these complex substrates were capable of replacing the trace elements and micronutrients typically provided by CGXII medium. However, the composition of trace elements in these substrates may not be optimal for terpene biosynthesis. While the addition of beneficial trace elements (i.e., Mg^2+^) is possible, the removal of undesired trace elements such as Mn^2+^ or chelating compounds from the substrate might be challenging. Additionally, the presence of endogenous terpenes, such as limonene in orange peels (Wikandari et al., 2015) may interfere with downstream processing by complicating the purification of a target terpene product such as *trans*-nerolidol. Although these hydrolysates represent a sustainable and cost-effective alternative to conventional carbon sources, substrate selection remains critical.

## Data Availability

The original contributions presented in the study are included in the article/Supplementary Material, further inquiries can be directed to the corresponding author.

## Abbreviations

CCD, central composite design; CDW, cell dry weight; DMAPP, dimethylallyl pyrophosphate; DoE, design of experiment; FPP, farnesyl pyrophosphate; GGPP, geranylgeranyl pyrophosphate; IPP, isopentenyl pyrophosphate; IPTG, isopropyl-β-D-1-thiogalactopyranoside; MEP, methylerythritol 4-phosphate; MVA, mevalonate; OFAT, one-factor-at-a-time; PDB, Plackett-Burman design; rDOS, relative dissolved oxygen saturation; RSM, response surface methodology.
