# Supplementary material for: Enabling and improving trans-nerolidol production by Corynebacterium glutamicum: combining metabolic engineering and trace elements medium refinement
Source: Front Bioeng Biotechnol. 2025 Jun 23;13:1621955. doi: 10.3389/fbioe.2025.1621955 (PMC12230084; doi:10.3389/fbioe.2025.1621955)
Supplement: Supplementary file 1 [file Datasheet1.PDF]

Supplementary Material

Supplementary Table S1: Oligonucleotides used in this work.

Oligonucleotide name	Sequence (5’ → 3’)	Purpose
1134	AATACGCAAACCGCCTCTCC	Forward sequencing primer for pECXC99E
1135	TACTGCCGCCAGGCAAATTC	Reverse sequencing primer for pECXC99E and pECXT-P_{syn}
1138	TCAGTGAGCGAGGAAGC	Forward sequencing primer for pECXT-P_{syn}
J003	ATGGAATTCGAGCTCGGTACCCGGGGAAAGGAGGCCCTTCAGATGGACTTTCCGCAGCAACTCG	Gibson assembly cloning of *ispA*$_{Ec}$-*NS*$_{Tw}$ operon into pECXC99E and pECXT-P_{syn}
J004	TTATTTATTACGCTGGATGATGTAGTCCGC	
J007	CATCCAGCGTAATAAATAAAACTGCCACACGAACGAAAGGAGGCCCTTCAGATGGCATTCTTCGGCTCCTCC	
J008	GCATGCCTGCAGGTCGACTCTAGAGTTACTTGATCAGGATGGACTTCAGGAACTCCTC	
J021	CGCAGCAACTCGAAGCC	Sequencing of *ispA*$_{Ec}$-*NS*$_{Tw}$ operon
J022	GACGCGGAAGGCAAACACG	
J029	CGTCTATCATCCCACTGAAGACCA	
J030	CATGGCTCTTTACGAAGCGAG	
J031	CCGAACTGCGTATCAACCTCA	

Supplementary Table S2: Trace element compositions used in this work. Concentration is given in g L^{-1} within the final medium.

Compound	BioLector/shake flask				Fermenter
	Standard	Steepest ascent	High MgSO4	Refined	Refined
$MgSO_4 \times 7\ H_2O$	0.25	0.8025	1.0	0.40125	8.025
$CaCl_2$	0.01	0.0099	0.01	0.01485	0.297
$FeSO_4 \times 7\ H_2O$	0.01	0.0121	0.01	0.00495	0.099
$MnSO_4 \times H_2O$	0.01	0.0126	0.01	0.0063	0.126
$ZnSO_4 \times 7\ H_2O$	0.001	0.00091	0.001	0.000455	0.0091
$CuSO_4$	0.0002	0.0002	0.0002	0.0002	0.004
$NiCl_2 \times 6\ H_2O$	0.00002	0.00002	0.00002	0.00002	0.0004

Supplementary Table S3: Setup and titer of the first CCD. Trace elements were prepared as a 100x concentrated stock solution each. The amount of each trace element solution that was added to 1 mL of media for the BioLector cultivation is given in µL. *trans*-Nerolidol titer is given in mg L^{-1}. Standard CGXII trace elements served as center point (12.2 ± 1.2 mg L^{-1}; n = 6).

Run	Stock solution added to each well [µL]					Titer [mg L^{-1}]
	$FeSO_4$	$MnSO_4$	$ZnSO_4$	$CaCl_2$	$MgSO_4$	
1	15	15	15	5	15	10,6
2	15	5	15	5	5	5,2
3	10	10	0	10	10	4,1
4	15	15	5	5	5	7,4
5	5	5	5	15	5	5,8
6	10	10	10	10	10	11,6
7	5	15	15	5	5	5,7
8	10	0	10	10	10	8,6
9	15	15	15	15	15	9,8
10	10	10	10	10	10	11,4
11	5	15	15	15	15	10,5
12	5	5	5	15	15	13,5
13	15	5	15	15	15	10,7

14	10	20	10	10	10	11,0
15	15	15	15	15	5	6,5
16	10	10	10	20	10	11,4
17	5	5	15	15	5	5,6
18	5	15	5	15	5	7,2
19	10	10	10	10	20	12,7
20	5	5	15	15	15	11,8
21	15	5	5	15	15	11,4
22	10	10	10	10	10	12,2
23	5	5	5	5	5	5,6
24	5	15	15	5	15	11,2
25	20	10	10	10	10	10,3
26	15	15	5	5	15	10,6
27	15	5	15	15	5	5,1
28	15	5	5	15	5	5,0
29	0	10	10	10	10	0,0
30	10	10	10	10	0	0,0
31	10	10	10	0	10	13,3
32	5	5	15	5	5	6,6
33	5	15	5	5	5	7,9
34	10	10	10	10	10	10,8
35	15	15	5	15	15	13,6
36	15	5	5	5	15	13,4
37	15	5	5	5	5	7,7
38	10	10	10	10	10	14,5
39	5	15	5	5	15	17,6
40	15	15	15	5	5	7,4
41	5	5	5	5	15	12,0
42	10	10	10	10	10	12,7
43	5	5	15	5	15	12,5
44	5	15	15	15	5	7,9
45	15	5	15	5	15	15,5
46	10	10	20	10	10	10,9

47	15	15	5	15	5	7,7
48	5	15	5	15	15	15,2

Supplementary Table S4: Effects of the trace elements on *trans*-nerolidol titer considering first order and quadratic effects. The effect of each factor, the F-values and their probabilities are given. Squared factors indicate quadratic effects.

**Factor**	***t*-value**	**Prob > *t***
Intercept	13.01	<0.001
$FeSO_4$	0.80	0.43
$MnSO_4$	0.99	0.33
$ZnSO_4$	-0.36	0.72
$CaCl_2$	-0.94	0.35
$MgSO_4$	8.43	<0.001
$FeSO_4$^2	-3.13	0.003
$MnSO_4$^2	-0.31	0.76
$ZnSO_4$^2	-1.70	0.09
$CaCl_2$^2	-1.27	0.21
$MgSO_4$^2	-2.39	0.02
	**F-value**	**Prob > F**
First Order	14.74	<0.001
Quadratic Effects	3.88	0.006
Lack of fit	3.41	0.087

Supplementary Table S5: Setup of steepest ascent. Trace elements were prepared as a 100x concentrated stock solution each. The amount of each trace element solution that was added to 1 mL of media for the BioLector cultivation is given in µL. *trans*-Nerolidol titer is given in mg L^{-1}. Standard CGXII trace elements were applied at distance 0. Experiments were performed in triplicates (mean ± sd).

Run	Distance	Stock solution added to each well [µL]					*trans*-nerolidol [mg L^{-1}]	
		$FeSO_4$	$MnSO_4$	$ZnSO_4$	$CaCl_2$	$MgSO_4$	Calculated	Measured
1	0	10.0	10.0	10.0	10.0	10.0	9.6	10.6 ± 1.1
2	0.5	10.2	10.3	9.9	9.7	12.5	11.1	12.3 ± 1.7
3	1.0	10.5	10.6	9.8	9.5	14.9	12.7	10.2 ± 1.8
4	1.5	10.7	10.9	9.7	9.2	17.4	14.2	12.3 ± 1.7
5	2.0	10.9	11.1	9.6	8.9	19.8	15.7	14.2 ± 0.8
6	2.5	11.2	11.4	9.5	8.6	22.3	17.3	9.7 ± 1.6
7	3.0	11.4	11.7	9.4	8.4	24.7	18.1	13.0 ± 0.6
8	3.5	11.6	12.0	9.3	8.1	27.2	20.4	12.8 ± 0.3
9	4.0	11.9	12.3	9.2	7.8	29.6	21.9	14,4 ± 1.6
10	4.5	12.1	12.6	9.0	7.5	32.1	23.4	17.1 ± 0.7
11	5.0	12.3	12.9	8.9	7.3	34.6	25.0	16.3 ± 1.4

Supplementary Table S6: Setup and titer of the second CCD. Trace elements were prepared as a 100x concentrated stock solution each. The amount of each trace element solution that was added to 1 mL of media for the BioLector cultivation is given in µL. *trans*-Nerolidol titer is given in mg L^{-1}. The trace elements composition of distance 4.5 of the steepest ascent experiment (Supplementary Table S5) served as center point (13.01 ± 0.76 mg L^{-1}; n = 6).

Run	Stock solution added to each well [µL]					Titer [mg L^{-1}]
	$FeSO_4$	$MnSO_4$	$ZnSO_4$	$CaCl_2$	$MgSO_4$	
1	4.95	18.90	4.55	14.85	16.05	13.1
2	12.10	12.60	9.10	19.80	32.10	11.3
3	12.10	12.60	9.10	9.90	32.10	11.6
4	12.10	25.20	9.10	9.90	32.10	13.3
5	18.15	6.30	13.65	14.85	16.05	12.1
6	12.10	12.60	9.10	9.90	32.10	13.5
7	18.15	18.90	13.65	14.85	16.05	12.3

8	4.95	18.90	4.55	4.95	16.05	13.4
9	12.10	12.60	9.10	9.90	0.00	0.0
10	18.15	6.30	4.55	14.85	45.15	11.3
11	18.15	18.90	13.65	4.95	45.15	11.8
12	24.20	12.60	9.10	9.90	32.10	12.8
13	12.10	12.60	9.10	9.90	32.10	13.0
14	18.15	6.30	4.55	4.95	45.15	14.0
15	12.10	12.60	9.10	9.90	32.10	14.1
16	4.95	18.90	13.65	14.85	45.15	12.3
17	4.95	18.90	13.65	4.95	16.05	10.6
18	18.15	6.30	4.55	4.95	16.05	14.1
19	18.15	18.90	4.55	14.85	45.15	13.8
20	4.95	6.30	4.55	4.95	45.15	15.0
21	4.95	18.90	4.55	14.85	45.15	14.7
22	4.95	6.30	13.65	14.85	45.15	12.5
23	12.10	12.60	9.10	0.00	32.10	13.4
24	4.95	6.30	4.55	4.95	16.05	12.8
25	12.10	12.60	18.20	9.90	32.10	12.1
26	4.95	18.90	13.65	14.85	16.05	12.8
27	18.15	18.90	4.55	14.85	16.05	14.1
28	18.15	18.90	4.55	4.95	45.15	14.8
29	18.15	6.30	4.55	14.85	16.05	14.6
30	4.95	18.90	4.55	4.95	45.15	8.9
31	0.00	12.60	9.10	9.90	32.10	0.0
32	4.95	6.30	4.55	14.85	45.15	14.2
33	12.10	12.60	9.10	9.90	64.20	13.7
34	12.10	12.60	0.00	9.90	32.10	4.5
35	18.15	6.30	13.65	4.95	16.05	13.0
36	4.95	6.30	13.65	4.95	45.15	13.6
37	4.95	6.30	13.65	4.95	16.05	13.5
38	12.10	0.00	9.10	9.90	32.10	14.1
39	18.15	18.90	4.55	4.95	16.05	16.5
40	4.95	6.30	13.65	14.85	16.05	14.3

41	12.10	12.60	9.10	9.90	32.10	12.7
42	18.15	6.30	13.65	14.85	45.15	12.4
43	12.10	12.60	9.10	9.90	32.10	13.2
44	18.15	18.90	13.65	14.85	45.15	16.9
45	4.95	6.30	4.55	14.85	16.05	17.0
46	18.15	6.30	13.65	4.95	45.15	15.6
47	4.95	18.90	13.65	4.95	45.15	14.1
48	18.15	18.90	13.65	4.95	16.05	14.9

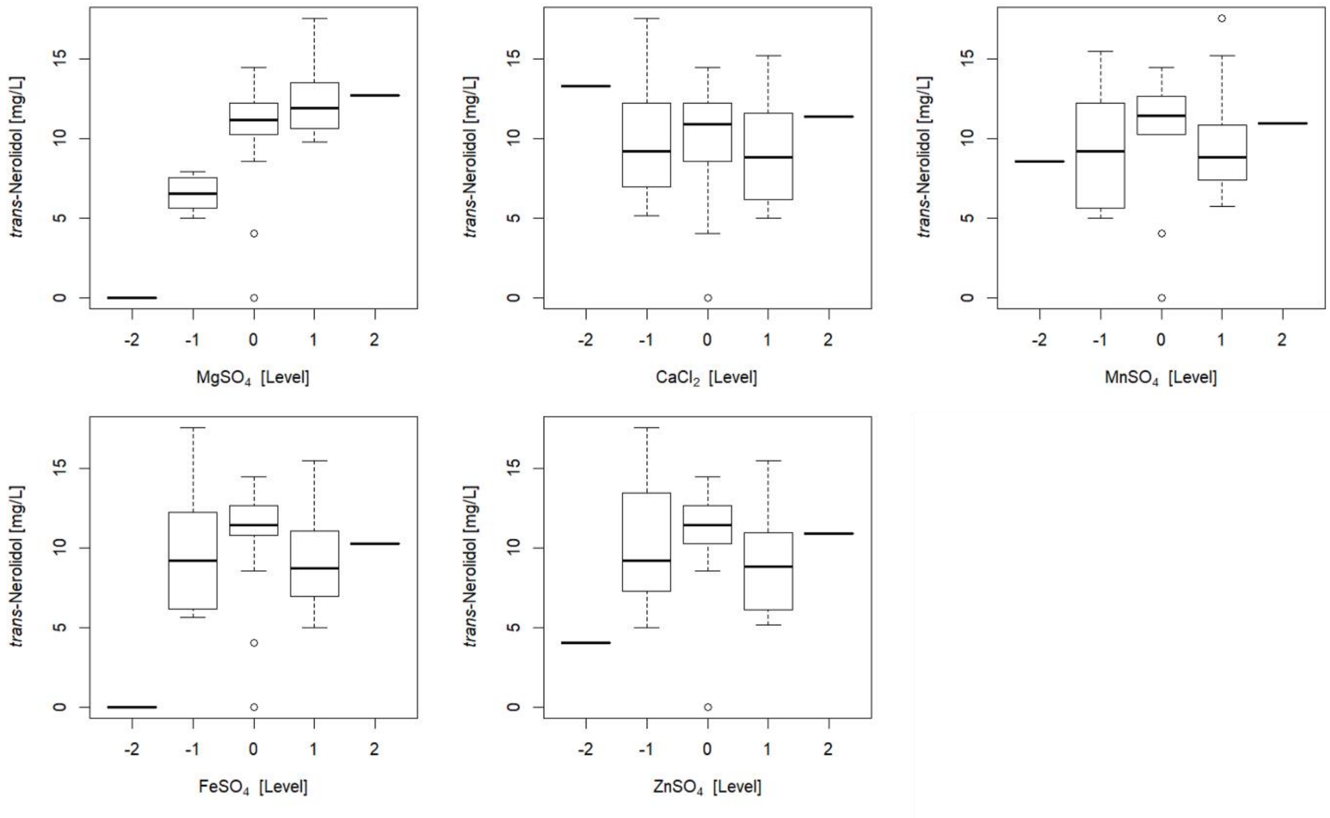

Supplementary Figure S1: Boxplots of the first DoE. Given are the *trans*-nerolidol titers for the five factors $MgSO_4$, $CaCl_2$, $MnSO_4$, $FeSO_4$, and $ZnSO_4$. Level 0 corresponds to the standard CGXII concentration of the respective trace element. Level -1 and 1 correspond to 50% and 150% and -2 and 2 to 0% and 200% of the standard CGXII concentration, respectively.
